# A Network-Based Mixed Methods Approach to Analyze Current Perspectives on Personalized Oncological Medicine in Austria

**DOI:** 10.3390/jpm10040276

**Published:** 2020-12-12

**Authors:** Ines Viktoria Stelzer, Anna Sierawska, Alena Buyx, Judit Simon

**Affiliations:** 1 Ludwig Boltzmann Institute Applied Diagnostics, General Hospital of Vienna, 1090 Vienna, Austria; judit.simon@meduniwien.ac.at; 2 Department of Health Economics, Center for Public Health, Medical University of Vienna, 1090 Vienna, Austria; 3 Institute for History and Ethics of Medicine, Technical University of Munich, 81675 Munich, Germany; anna.sierawska@tum.de or a.buyx@tum.de (A.B.); 4 QUEST Center, Berlin Institute of Health, 10178 Berlin, Germany

**Keywords:** personalized medicine, oncology, molecular pathology, experts, patients, qualitative interview study, mixed methods approach, network theory, Austria

## Abstract

Personalized medicine (PM) to tailor healthcare (HC) to the individual, is a promising but challenging concept. So far, no study exists investigating stakeholders’ perspectives on PM in oncology in Austria potentially hindering implementation, which was the aim of this study. We performed semi-structured interviews among experts (*n* = 14) and cancer patients (*n* = 2) of the Vienna General Hospital and the Medical University of Vienna and analyzed them by a mixed methods network theoretical approach. Study results show a great variety of topics addressed by the interviewees. Clear differences in the topic selection between patients and experts could be observed. Patient-doctor relationship was the most prominent theme among experts, whereas HC systems and public health in PM was more relevant for the patients. Although promising new molecular pathology methods were explicitly mentioned, the experts believed that their practical implementation and the implementation of PM in standard care will take a long time in Austria. A variety of concerns regarding PM were mentioned by the experts, including communication issues and knowledge gaps. Besides important insights into the current situation of PM in Austria, the study has shown that network theory is a powerful tool for analyzing qualitative interview data.

## 1. Introduction

Personalized medicine (PM) is defined as a medical concept—aiming for providing targeted disease prevention, predisposition determination and therapeutic strategies in a timely manner based on individuals’ phenotypes and genotypes [1]—and has the potential to revolutionize healthcare (HC). The idea of overcoming the traditional one-size-fits-all approach by offering individualized care to a patient on the one hand, avoiding late diagnoses, unnecessary treatments, their costs and side effects in non-respondent groups on the other hand is tempting and could lead to an improved allocation of health resources, better outcomes, an increase in patients’ quality of life and a reduction of health expenditures, thus leading to purposeful and cost-effective HC strategies and optimized HC systems [2,3,4,5]. Especially in the field of personalized oncological medicine (POM) remarkable progress has been made in recent years, in particular resulting from the use of powerful biomarkers and molecular pathology (MP) methods [5,6,7,8,9]. Despite of all these advances having been made, several translational, ethical, societal and (health) economic challenges with respect to PM have been identified. These include the risk of exploding HC costs which might be dedicated to a small group of people only [10], the lack of standardized procedures regarding evidence generation, health economic evaluations, health technology assessments (HTA) and reimbursement processes [9,11,12], the need of new trial designs, in turn leading to ethical challenges [13], the risk of health inequity and data manipulation, the complexity of interpreting sequencing results and decision making both from HC professionals’ and patients’ perspective and implementation challenges, bringing new PM and MP techniques—such as genomic services—into clinics [14]. 

In order to guarantee the successful implementation of POM HC strategies in clinical practice and HC systems, as well as their acceptance in the society, the early involvement of stakeholders and an open anticipatory discourse on potential issues is essential. Interview studies—reflecting stakeholders’ perspectives and views—may help to get insight into potential risks, fears and research gaps. Thus, arising challenges can be communicated to decision and policy makers to be addressed at an early stage, accelerating the realization of PM and HC systems’ preparation to it. In the study by Budin-Ljøsne et al. [15] for example, semi-structured telephone interviews among patient and interest organizations’ representatives in Europe and North America were carried out to investigate their views on PM. Stakeholders of the German HC system—including amongst others patient organizations, clinicians, researchers, representatives of the pharmaceutical industry and regulatory authorities [16], and clinical researchers working on PM in colorectal cancer [17]—were asked to share their thoughts on the new medical concept. The former study focused in particular on ethical issues and the latter on patient-doctor interactions. Further qualitative studies involving patients with various types of cancer were performed to analyze their understanding of PM, as well as their experiences and expectations with respect to gene expression analysis and next generation genomic sequencing in German [18], Canadian [19] and North American [20] institutions. 

Although existing studies give valuable insight into the general situation of PM, the single results have to be put into perspective regarding their transferability to other countries, as they might reflect national HC systems’ specifics. Thus, studies with a similar focus but being performed in different countries are of high importance. To the best of our knowledge no interview study investigating Austrian stakeholders’ perspectives on PM has been performed so far. The aim of this study is to close this gap, by analyzing experts’ and cancer patients’ views on ethical, (health) economic, societal and translational aspects with respect to POM specifically focusing on MP examples. Experts and patients were selected as interviewees as they represent an important and highly affected group of stakeholders. The former group is strongly involved in new developments and their implementation processes, therefore their expertise and experience are important for further research and strategic development and the latter group represents those individuals that should benefit the most from PM. 

In contrast to the studies mentioned above, that were analyzed by standard qualitative methods such as content analysis, a mixed methods approach based on network theory was used to quantify and visualize the answers and topics addressed by the study participants and to identify underlying patterns or interlinks among them. Network theory is a graph-theoretical approach for analyzing interactions between objects, by representing them as so-called nodes that are connected by a link if an interaction of a specified type occurs between them [21]. Bipartite networks are certain network types, whose nodes can be partitioned in two disjoint node sets or node partitions, so that only links between the two node partitions exist [21,22]. Networks and specifically bipartite networks have been used in a variety of fields, including biology, medicine [22] and social sciences [23] to extract information from data structures. The investigation of qualitative interview data by a mixed methods approach—defined as the use of both quantitative and qualitative data and methods [24] and being known as a powerful tool in health services research [25]—is not new. However, the concept of applying network theory to this kind of data has only recently been introduced [26] and implemented [27,28,29]. Due to a stronger focus on the interactions between interviewees and the addressed topics— compared to the examples given in the study by Pokorny et al. [26]—another network structure has been chosen and different methods for analyzing network properties have been applied in this study. Additionally, topics selected according to a specific scheme were visualized and described with respect to their content, revealing concerns about POM in a transparent way. Thus, the study does not only give insight into the structure of the addressed topics, but also represents stakeholders’ current views on POM in Austria and encourages the application of network theoretical methodology to interview data. 

## 2. Materials and Methods

The methodology applied in this study follows a mixed methods approach—analyzing qualitative interview data by the use of network theory—that will be described in the following subsections. The patient and family interview study with the study number 2004/2018 has been approved by the Ethics Commission of the Medical University of Vienna (MUV) on 10 December 2018, that also confirmed that no ethics approval is needed for carrying out the expert interviews. All study participants gave their informed consent before they were interviewed. Personal data were pseudonymized and could only be accessed by the research team. They are not publicly available for privacy reasons. The study was conducted in accordance with the Declaration of Helsinki and complied with the General Data Protection Regulation and the Austrian adjustment standards as amended.

### 2.1. Qualitative Data Collection

For the qualitative data collection process, semi-structured interviews were carried out by AS. The underlying interview topic guide—developed by AS—was based on an extensive literature search on challenges in PM and was slightly adapted in the course of the individual interviews. Two groups of experts—namely clinicians and health system experts on the one hand and basic scientists on the other hand—being experienced in POM and working at the MUV or the Vienna General Hospital—which is an academic tertiary referral hospital and among Europe’s largest hospitals—were invited to participate in the study. Patients suffering from cancer—being treated within the hospital and not being younger than 18 years or older than 80 years—were eligible for participation in the interview study. Recruitment took place via recommendation and direct approach. The interviews were planned for an average duration of 45 min. The open-ended questions for discussion addressed in the interview guide referred to knowledge, experience, expectations, challenges and opinions with respect to POM. Specifically, the topics decision-making, HC systems/public health, data collection and patient-doctor-relationships were included. Experts were additionally asked to reflect on (research) ethics, (health) economics, clinical, translational and communication aspects and the transfer of knowledge in this context. Moreover, clinical vignettes—representing clinical scenarios—were created and discussed with the patients. The in-depth interviews were audio-recorded, transcribed verbatim and cross-checked. Using the qualitative data analysis software MAXQDA 2018.2, thematic analysis [30] was performed by AS which means that individual interview phrases—that is answers given by the interviewees—matching the topics represented in the interview guide, were categorized into codes and then aggregated into higher-level themes. 

### 2.2. Data Analysis and Visualization

In order to explore the relations between the interviewees and the addressed codes, a bipartite interview network 𝒩 was created, where both the interviewees (patients and experts) and the codes were represented as nodes from two different node partitions, that were linked if an individual responded to a certain code, i.e., if interview phrases forming the code—in the course of thematic analysis described in Section 2.1—were mentioned by the individual. Inversely, if no link between an individual and a specific code occurred in the interview network, this meant that the individual did not talk about this topic during his or her interview. To this end, the qualitatively collected interview data were converted into the adjacency matrix *A*, being the mathematical representation of a network [21]. More precisely, the binary incidence matrix *B* was defined, with the columns representing the individual interviewees and the rows representing the identified codes. If an individual *j* responded to a code *i*, the matrix entry *b_ij_* was set equal to 1 and to 0 otherwise—i.e., expressed mathematically—

$$b_{ij}:=\{\begin{array}{l} 1,\;\;\;\;\;\;\;\;\;if\;individual\;j\;responded\;to\;code\;i\\ 0,\;\;\;\;\;\;\;\;\;if\;individual\;j\;did\;not\;respond\;to\;code\;i \end{array}$$

for i ϵ C:={1,…,M} and j ϵ I:={1,…, N}, where *M* denotes the number of codes and *N* the number of interviewees. The adjacency matrix *A* is then defined as the following (*M + N*) × (*M + N*) block matrix

$$A:\;=\;\left(\begin{array}{cc} 0_{M\times M} & B\\ B^{T} & 0_{N\times N} \end{array}\right),$$

where *B^T^* denotes the transpose of *B* and 0*_M×M_* and 0*_N×N_* denotes the *M* × *M* and the *N* × *N* zero matrix respectively. This matrix has been used for visualizing the bipartite interview network 𝒩. Furthermore, network properties such as the nodes’ degrees were calculated. The degree of a node is defined as the number of links attached to it [21], therefore quantifying the number of codes addressed by the interviewees and the number of interviewees responding to a certain code in our specific case. Mathematically, for the *M + N* nodes of the bipartite interview network, their degrees can be calculated as the corresponding column or row sum of the matrix *A*:

$$k_{l}:=\sum_{j=1}^{M+N}a_{lj}=\sum_{i=1}^{M+N}a_{il},\;l\;\epsilon \;\left\{1,\ldots ,\;\left(M+N\right)\right\}.$$

Additionally, subnetworks of the interview network were identified, in which all nodes of both node partitions are linked to each other and are of maximal size. These so-called maximal bicliques or concepts [31] are the structurally strongest communities among bipartite networks [32] and show which codes were addressed together by the same interviewees and which interviewees focused on the same topics, therefore revealing patterns among the given responses.

Per code, the individual interview phrases given by each study participant responding to this code, were checked for a positive or negative key statement regarding the existence of a concern and classified into “yes” and “no” accordingly. The option “yes” was negatively connoted meaning that concerns or issues were mentioned by the interviewees. Codes and single interviewees’ responses where such dichotomous classification was not feasible—that is where no statement on concerns or no concerns occurred in the corresponding interview phrases—were excluded. The data could be transformed into a so-called multigraph network denoted as 𝒩_𝒞_ —i.e., a network in which the same two nodes can be connected by more than one link—where the codes represented one node partition and the options “yes” and “no” the other node partition. The individuals’ yes or no responses were represented as links to the corresponding codes. The links’ line widths corresponded to the number of positive and negative answers, thus highlighting the number of concerns and issues identified or not identified by the study participants, as well as controversial opinions. Mathematically, the corresponding adjacency matrix *A*′ had the same structure as matrix *A*, however, the *M*′*_×_ 2* incidence matrix *B*′ was defined as follows:

$$b_{i1}^{\prime }:=\#y_{i},\;b_{i2}^{\prime }:=\;\#n_{i},\;i\;\epsilon \;\left\{1,\ldots ,M^{\prime }\right\},$$

where *M*′ denotes the number of codes and *#y_i_* and *#n_i_* the number of yes and no answers of all interviewees for code *i*, assigning weights to the links according to the number of answers.

The analysis was performed using the statistical software R 4.0.2 [33]—including the ggplot package [34] and the biclique package v1.0.5 [35]—as well as the network analysis and visualization tool Gephi 0.9.2 [36].

## 3. Results and Discussion

### 3.1. Description of the Qualitative Interview Data

Altogether 16 stakeholders—i.e., 14 experts and two cancer patients—were interviewed from 8 December 2018 to 21 February 2019. The sample size was in a magnitude comparable to similar studies [16,17] and large enough to guarantee data saturation. Amongst the experts, 10 were clinicians or health system experts and four were basic scientists. They had different specializations, including urology (five individuals), oncology (one individual), pathology (two individuals), nuclear medicine (four individuals), public health (one individual), health economics (one individual), clinical pharmacology (one individual) and surgery (one individual). Some experts had more than one specialization, therefore the number of experts per specialization category does not sum up to the total number of experts. The experts’ gender distribution was 79% men and 21% women. One of the patients was female and one was male. All interviews took place face-to-face and ranged from 42–80 min. In total 43 codes were identified during the interview coding process that formed the following seven themes: 1. Ethics and PM, 2. Decision-making and PM, 3. Effectiveness evidence and PM, 4. Interpretation of PM, 5. HC systems/public health and PM, 6. Challenges in PM and 7. Patient-doctor relationships and PM. The codes belonging to the corresponding themes can be found in Table 1. On average a theme consisted of six codes. Theme “2. Decision-making and PM” had nine and therefore the most codes, theme “4. Interpretation of PM” had the minimum number of four codes. Two codes explicitly referring to MP—namely “2.2 (Concerns about) liquid biopsies” and “5.1 Views on/Concerns about genetic screening w.r.t. the public”—were identified, the former having been mentioned in connection with theme “2. Decision-making and PM” and the latter with “5. HC systems/public health and PM”.

### 3.2. Network Analysis of the Qualitative Interview Data

**Figure 1:**
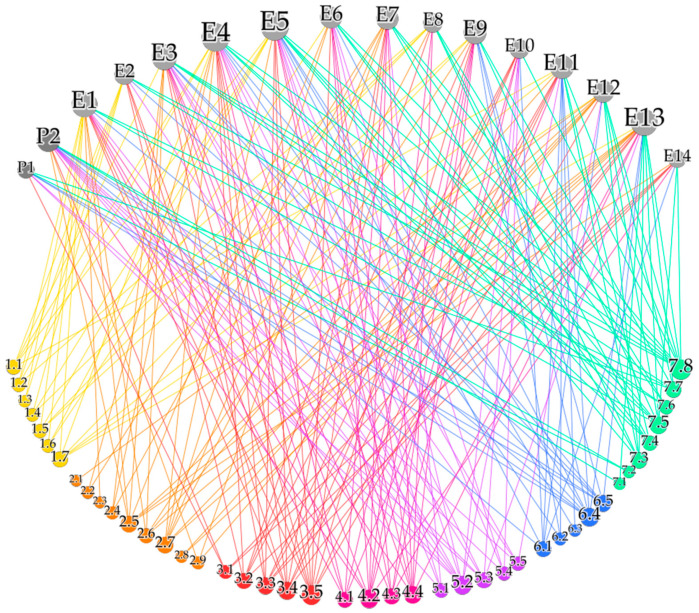
Visualization of the interview network 𝒩 showing the interviewees (gray nodes at the top) and the codes addressed (colored nodes at the bottom) represented as links.

**Figure 2:**
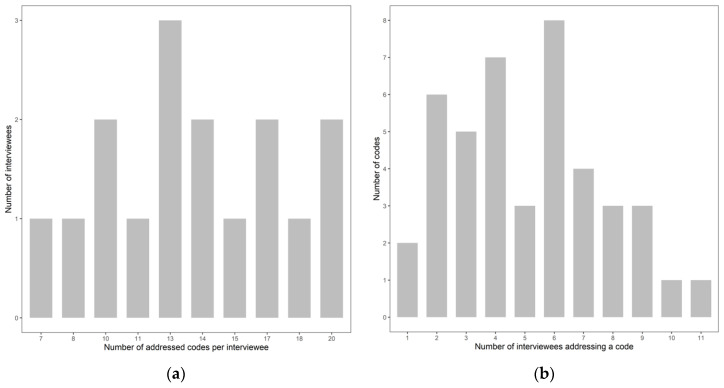
Degree distribution showing (**a**) the number of addressed codes per interviewee (*x*-axis) and the number of interviewees addressing this specific number of codes (*y*-axis) and (**b**) the number of interviewees addressing a code (*x*-axis) and the number of codes addressed by this specific number of interviewees (*y*-axis).

The qualitative interview data can be transformed into a bipartite interview network 𝒩—visualized in Figure 1—where the 16 interviewees represent one node partition and the 43 codes the other node partition. Interviewees were illustrated as gray nodes and named as {P_1_, P_2_} representing the patients and {E_1_, …, E_14_} representing the experts. The codes were named according to their numbering in Table 1—i.e., {1.1, …, 7.8}—and were colored subject to the theme they belonged to. Whenever a patient or an expert addressed a specific code during the interview, this was represented as a link between the corresponding nodes, colored according to the code node’s color. This resulted in 220 links, that is responses given by the interviewees. The size of a node in Figure 1 corresponded to its degree or equivalently to the number of links attached to it. Thus, interviewees responding to more codes and codes being addressed more often by the interviewees appeared bigger in the interview network. This shows that the two experts E_4_ and E_5_—both being clinicians or health system experts—addressed the highest number of codes, whereas patient P_1_ responded to the lowest number of codes. Inversely, the largest code node in Figure 1 shows the highest response rate to code “7.8 (Concerns about) best decision-making” and the lowest number of responses for “2.1 Views on HTA” and “2.3 Ethics and economics”. The exact number of codes and themes responded to by the interviewees—separately shown for patients and experts—is summarized in Table 1. Since the addressed codes and themes represent the topics included in the interview guide, differences in the number of patient and expert responses can be observed, resulting from the extended expert interview guide. On the other hand, patients shared their views on health economics (code 2.5) and preclinical studies (code 3.1) for example, although these aspects were not included in their interview guide but arose during the semi-structured interviews. Additionally, the degree distribution represented as bar plots in Figure 2 gives an overview on the number of addressed codes per interviewee (Figure 2a, *x*-axis) and the number of interviewees that addressed this specific number of codes (Figure 2a, *y*-axis) as well as the number of interviewees addressing a code (Figure 2b, *x*-axis) and the number of codes that were addressed by this specific number of interviewees (Figure 2b, *y*-axis). Thus, it becomes visible from Figure 2a that two individuals contributed to the maximal number of 20 codes and therefore addressed 47% of the 43 codes, whereas the average number of codes responded to by the study participants was 14. These individuals were the two experts E_4_ and E_5_ as identified by their node size in the interview network in Figure 1. On average a code was addressed by five individuals. Figure 2b shows that one code was responded to by 11—and therefore almost 70% of the—interviewees. This prominent code was 7.8—considering its node size in Figure 1—and was exclusively addressed by the experts and by 80% of both expert groups. In general, theme 7 had the most responses among the experts, followed by theme “3. Effectiveness evidence in PM” and “2. Decision-making and PM”. Codes with a high expert response rate of at least eight responses—in addition to code 7.8—were “3.4 Views on evidence”, “3.5 Views on/Concerns about RCTs”, “4.2 Various understandings of PM”, “4.4 Overdiagnosis” and “7.5 (Concerns about) communication”. As mentioned above, the two codes “2.1 Views on HTA” and “2.3 Ethics and economics” were only addressed by one expert each. This might be due to the fact that HTA is a relatively new field in Austria and does not have the same importance in HC-related decision-making compared to some other European countries. On the other hand, 50% of the experts uttered health economics concerns (2.7), although it could have been expected that topics on costs or health economics are less relevant in Austria due to the dominantly social health insurance-based HC system with full coverage of the population. No prominent differences in the number of codes addressed by the two expert groups could be observed, apart from “2.6 (Concerns about the) role of pharmaceutical industry”, “6.5 (Concerns about) consent for data collection” and “7.7 (Concerns about) incidental findings”, having been responded to by 75% of the basic scientists and by only 20% of the clinician and health system expert group. Regarding code “3.4 Views on evidence” it was the other way around with 70% responses of the clinical and health system expert group and 25% responses by the basic scientists.

Although “HC systems/public health and PM” was the least often addressed theme by the experts, it was the most relevant topic for the patients according to the number of responses. This indicates that different topics were paid attention to by different groups of stakeholders. However, due to patients being an underrepresented group among the interviewees, further research would be needed to investigate such an effect and to confirm the importance of the topic for patients in general. Nevertheless, these results show that aspects referring to the Austrian HC system and public health receive less attention by practicing clinicians and basic scientists, despite of their relevance in PM. Codes of high interest by both the experts and the patients were “5.2 (Concerns w.r.t.) the Austrian context” and “6.4 (Concerns about) big data collection and use”. The two MP-specific codes 2.2 and 5.1 were addressed two and four times respectively. This reflects well the heterogeneous background of the interviewed persons and the specificity of the topic area.

**Figure 3:**
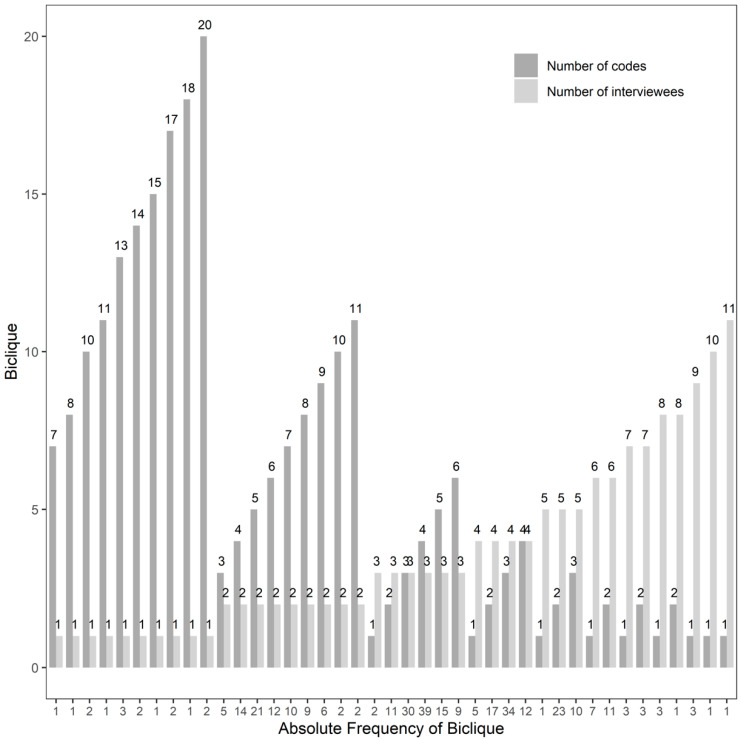
Overview on the identified bicliques, summarizing the number of codes having been addressed by a specific number of interviewees and their frequency.

In order to investigate whether specific groups of people addressed specific groups of codes, the interview network’s bicliques were extracted. Figure 3 summarizes the identified subnetworks, consisting of a certain number of codes having been addressed by a specific number of interviewees and their frequency. Considering the high number of bicliques with a low number of codes and/or interviewees in Figure 3, it can be concluded that a large variety of topics was selected by the single study participants. The largest bicliques with respect to the number of interviewees and codes identified consisted of six codes addressed by the same three study participants (nine different combinations found), three codes addressed by the same five interviewees (10 different combinations found) and four codes responded to by the same four interviewees (12 different combinations found). Among these bicliques, clinicians and health system experts addressing the codes “3.5 Views on/Concerns about RCTs”, “4.4 Overdiagnosis”, “6.4 (Concerns about) big data collection and use”, “7.5 (Concerns about) communication” and “7.8 (Concerns about) best decision-making” were most often observed. This, however, is not surprising considering the overrepresentation of this expert group and the high frequency of the identified codes. Thus, no prominent pattern among the responses given could be identified which might result from the heterogeneity of study participants or the interview design in general, covering a broad variety of topics.

**Figure 4:**
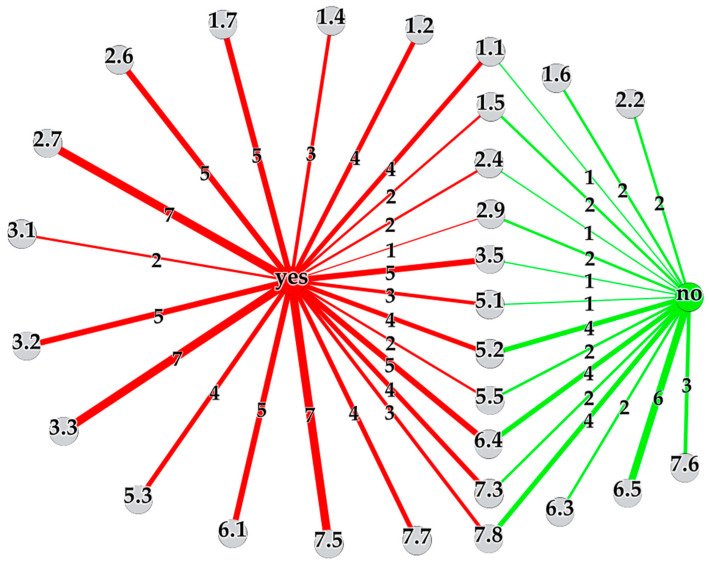
Visualization of the multigraph network 𝒩_𝒞_ showing the number of interviewees mentioning concerns/issues (links to red “yes”-node) or no concerns/issues (links to green “no”-node) for selected codes (gray nodes).

For 28 out of the 43 codes, the interviewees’ answers could be classified into “yes” or “no”, where “yes” meant that concerns or issues were uttered by them and “no” meant that this was not the case. From the 28 selected codes, 22 interviewee responses had to be excluded as they did not fit into the specified scheme. The results were summarized as the multigraph network 𝒩_𝒞_ in Figure 4, visualizing the selected codes as gray nodes and the two answer options “yes” and “no” as red and green node respectively. The number of yes and no answers for a specific code were represented as a link between the corresponding nodes, where the link’s line width corresponded to the number of answers, also shown alongside the link. Thus, the wider a link between a certain code and the “yes”/“no”-node appeared in the network, the more interviewees mentioned concerns/no concerns. As visible in Figure 4, only five codes (1.6, 2.2, 6.3, 6.5 and 7.6) with no concerns mentioned were detected, 11 codes led to controversial answers and for the remaining 12 codes all interviewees identified issues. In the following the main findings and concerns are summarized, referring to the experts’ interviews if not stated otherwise.

#### 3.2.1. Ethical Concerns

A variety of ethical concerns with respect to PM were identified. Although the importance of ethics was non-controversial (1.6), all respondents saw ethics approvals (1.2) and the role of ethics committees (1.4) critically, as the general feeling was that the process for getting an ethics approval is very time-consuming and that the ethics committee cannot always judge on the individual studies but is too strict in its evaluations in general. This statement indicates that the experts feel hindered in their work and research by the committee’s decisions made, which of course affects the future progression of PM. Thus, ethics committees play a central role in the development of PM HC strategies, deciding on ethically highly delicate topics. Specific ethics training of HC professionals as suggested in [4] on the one hand and transparency as well as information exchange regarding both the (processes for) decisions made by the ethics committees and the needs of the researchers and clinicians on the other hand might be helpful to accelerate the realization of PM based on studies with ethics approvals. Most of the interviewees, namely four out of five, furthermore uttered concerns over informed consents (1.1). Specifically, it was questioned whether patients would understand what would happen to their data being analyzed by MP-techniques in the era of PM. Fifty percent of the experts identified issues with ethics approvals (1.5) and one of them had concerns regarding the retrospective analysis of genetic patterns of stored tissue samples, where the two other interviewees said that this is not an issue at all, being one of the conflicting statements. All respondents identified ethical issues in PM in the course of the interviews (1.7), including the risk of resource limitations, the potentially unnecessary collection of personal data, the use of human cells in animals, the fact that findings on patients’ mutations might also affect family members, the risk of overdiagnosis in epigenetics and the pressure of clinicians to include patients into clinical trials for professional advancement, coming along with wrong promises conveyed to them although knowing that the individual study participant will not be able to benefit from the PM research results anymore.

#### 3.2.2. Concerns about Progress in Cancer Research

The latter ethical concern on wrong expectations and promises relates to the views on the progression of PM. All interviewees—including one patient—agreed that the progress in cancer research could be improved (6.1). Although molecular testing is commonly performed for specific cancer entities in Austria, the provision of POM treatment strategies based on this information is strongly limited. It was expected by the experts that the progress and the realization of PM HC will take a long time. This is in line with the patient and interest organizations’ expectations explored in [15], the disappointments of therapy limitations by clinical researchers identified in [17] and the physicians’ relativization of immediate benefits of PM testing methods in [19].

#### 3.2.3. Concerns about Data Collection

A hundred percent of respondents—including both patients—gave their consent regarding the collection of personal data in PM, under the condition that these data would only be used for medical purposes, their access would be restricted and individual data would not get published (6.5). Additionally, the storage of data was not seen as an issue per se (6.3). Despite of these answers, the study participants were split with respect to their views on potential concerns over real-world big data collection and use (6.4). Five out of nine respondents—including both patients—i.e., 56% were worried about access to the data by the wrong institutions—such as health insurance companies—which might be disadvantageous for patients in the future. Moreover, although the importance of data collection and use was undoubted in general, uncontrolled collection of personal information, the lack of training personal on how to handle data and data quality were questioned. Those four individuals that did not mention concerns, argued, that people also give away their (genetic) data to any companies day-to-day without knowing what actually happens to them. Regarding genetic information, one expert additionally said that the number of people being able to interpret these results is also limited. Focusing on the explicit example of genetic screening (5.1), three out of four people—including one patient—identified concerns, such as that knowing about mutations might lead to fear among patients and that they might ask for unnecessary treatment, which is both an ethically and health economically relevant issue. Awareness training, strict access restrictions and a secure data infrastructure would be required to dispel or minimize these concerns.

#### 3.2.4. Concerns about Costs and Health Economics

All interviewees mentioned health economic (2.7) and almost all—i.e., two out of three—cost concerns (2.4), although their answers often did not coincide with respect to cost saving potentials in PM. Some study participants were convinced that overall costs will be saved even though there may be initial cost increases during the development process or that money will be simply used in a different way. One interviewee thought that biomarkers might reduce costs by identifying patients who will need follow-up therapies, another was skeptical though, because of the risk of overdiagnosis and the lack of information regarding their effectiveness. Nevertheless, there was no doubt on liquid biopsies being high potential and cheap biomarkers for future decision-making, albeit with limited current use in daily clinical practice (2.2).

#### 3.2.5. Concerns about RCTs

The majority of respondents—that is 83%—mentioned concerns regarding the use of RCTs in PM (3.5). Two experts thought about the possibility of the development of effective PM drugs having been stopped in the past because of wrong trial designs or a too low number of treatment respondents. The interviewees were aware of new procedures in PM such as basket trials, umbrella trials or adaptive licensing, however it was not clear how to prove evidence of a treatment working in one patient only or how to take into account patients’ long and complex treatment histories.

#### 3.2.6. Concerns about Knowledge Gaps, Translational Issues and Communication Issues

All interviewees addressing code 3.2, 3.3 and 7.5 mentioned concerns regarding existing knowledge gaps, translational and communication issues in PM. According to the interviewees, knowledge gaps occur between physicians of different hospitals and departments, within the medical field due to a lack of clinicians’ knowledge on data handling or molecular biology to give an example, between physicians working in research and practitioners and between experts and the general public. This coincides with the results of the study by Sperber et al. [14], where the improvement of clinicians’ knowledge and acceptance of genomic medicine was found to be important for its successful implementation. As translational issues the transfer of biomarkers from basic research into clinical practice and communication issues between scientists and clinicians as well as between researchers/physicians and patients were mentioned, resulting in a lot of circulating misinformation. The hype on PM in the media and the missing possibility of the general public to access information sources or judge on their quality was believed to intensify this effect.

#### 3.2.7. Concerns about Patient-Doctor Relationships

Especially the patient-doctor communication—which will become more complicated as PM evolves—should be improved, considering that 67% of the experts uttered concerns about patients’ willingness or ability to participate in the decision-making process (7.3). Although it was emphasized that this is strongly dependent on the individual’s educational level and health literacy, potential reasons for patients’ preference for non-participation were the complexity of PM technologies, the lack of medical knowledge or not being willing to have the decision responsibility. A comparable result was found in [17], where German clinicians pointed out the difficulty of patients to understand complex PM therapy procedures. All interviewees said that the patient-doctor relationship is very important and expected it to play an even higher role in PM (7.6).

In order to address these challenges, the communication between stakeholders being involved in PM implementation processes has to be improved and specific trainings have to be provided. Thus, awareness of neglected topics—such as public health and HC system aspects among clinicians and basic scientists—can be raised and transparency in the decisions made by ethics committees can be expected. The specific training of HC professionals to increase their knowledge on MP methods, is seen as a crucial factor for realizing future PM HC strategies. Furthermore, also limitations of PM should be highlighted and communicated to the public, relativizing its progress to avoid false hopes. Communication tools used in the patient-doctor interaction should be developed guaranteeing the best possible involvement of patients in the decision-making process. In particular, patients should be educated and trained in understanding PM. Further evidence on the effectiveness and the costs of PM HC strategies is required, necessitating the collection of big data. Transparency on the use of these personal data and their protection should be guaranteed to avoid data abuse.

This study provides an extensive overview on user stakeholders’ perspectives on various key topics in PM and is the first of its type in Austria. It showed that the interviewees were aware of a variety of challenges in PM. However—due to the Vienna General Hospital being an academic tertiary referral hospital—the views of the clinical experts might reflect the hospital’s specifics and its strong research focus and might hinder its generalization for Austria. Another limitation of this study is the number of included patients that was very low, which might affect the conclusions. Additionally, women and basic scientists were an underrepresented group among the experts. Future studies, addressing these limitations would be needed to gain further insight into these specific groups’ perspectives on PM in Austria in addition to non-user stakeholder perspectives (e.g., decision-makers, funders). From the methodological point of view, network theory has shown to be a powerful tool for analyzing and visualizing the qualitative interview data, giving insight into topics being more and less relevant for the interviewees, showing patterns among the responses and highlighting the most controversial aspects. Reducing the data to the certain structure, however, leads to some loss of information. This applies to the dichotomization of the interviewees’ responses and the descriptive summary of their content. As with the classification and coding—being subjective to a certain degree—only a subset of responses can be investigated. Therefore, the suggested methodology is considered to be a complementary approach to the standard qualitative analysis being subject to future research and can add to it by identifying the most important topics and their interlinks.

## Figures and Tables

**Table 1:** Summary of the addressed codes and themes (number of patient/expert responses in brackets).

Theme	Code
1. Ethics and personalized medicine (PM) (0/27)	1.1 (Concerns about) informed consents (0/5) 1.2 Concerns about ethics approvals (0/4) 1.3 Ethical concerns (0/2) 1.4 Views on/Concerns about ethics committees (0/3) 1.5 (Issues w.r.t.) studies with ethics approvals (0/4) 1.6 (Concerns about the) importance of ethics (0/3) 1.7 Ethical issues (0/6)
2. Decision-making and PM (2/29)	2.1 Views on health technology assessment (HTA) (0/1) 2.2 (Concerns about) liquid biopsies (0/2) 2.3 Ethics and economics (0/1) 2.4 Cost concerns (0/3) 2.5 Views on health economics (1/6) 2.6 (Concerns about the) role of pharmaceutical industry (0/5) 2.7 Health economic concerns (0/7) 2.8 Lobbying (0/2) 2.9 (Concerns about) private versus public (1/2)
3. Effectiveness evidence and PM (2/33)	3.1 (Concerns about) preclinical studies (2/2) 3.2 Translational issues (0/6) 3.3 (Concerns about) knowledge gap (0/7) 3.4 Views on evidence (0/8) 3.5 Views on/Concerns about randomized controlled trials (RCTs) (0/10)
4. Interpretation of PM (1/28)	4.1 Views on hype (0/6) 4.2 Various understandings of PM (1/8) 4.3 Examples of personalized treatment (0/6) 4.4 Overdiagnosis (0/8)
5. Health care (HC) systems/public health and PM (7/20)	5.1 Views on/Concerns about genetic screening w.r.t. the public (1/3) 5.2 (Concerns w.r.t.) the Austrian context (2/7) 5.3 (Concerns about) access limitation to treatment (2/4) 5.4 Public health (1/3) 5.5 (Concerns about the) importance of preventive medicine (1/3)
6. Challenges in PM (5/22)	6.1 Views on/Concerns about progress in cancer research (1/5) 6.2 Improving understanding in society (0/3) 6.3 (Concerns about) data storage (0/2) 6.4 (Concerns about) big data collection and use (2/7) 6.5 (Concerns about) consent for data collection (2/5)
7. Patient-doctor relationships and PM (4/40)	7.1 Expertise (assessment) (1/1) 7.2 Decision-making patients’ perspective (2/0) 7.3 Concerns about patients’ participation in the decision-making process (0/6) 7.4 Shared decision-making (0/5) 7.5 (Concerns about) communication (0/8) 7.6 (Concerns about the) importance of doctor-patient relationships (0/4) 7.7 (Concerns about) incidental findings (1/5) 7.8 (Concerns about) best decision-making (0/11)
